# A Spatial Shape Constrained Clustering Method for Mammographic Mass Segmentation

**DOI:** 10.1155/2015/891692

**Published:** 2015-02-08

**Authors:** Jian-Yong Lou, Xu-Lei Yang, Ai-Ze Cao

**Affiliations:** ^1^School of Electrical Engineering, Xi'an Jiaotong University, Xi'an, Shaanxi 710049, China; ^2^Department of Computing Science, Institute of High Performance Computing, A^*^STAR, Singapore 138632; ^3^Department of Psychiatry, Vanderbilt University Medical Center, Nashville, TN 37232, USA

## Abstract

A novel clustering method is proposed for mammographic mass segmentation on extracted regions of interest (ROIs) by using deterministic annealing incorporating circular shape function (DACF). The objective function reported in this study uses both intensity and spatial shape information, and the dominant dissimilarity measure is controlled by two weighting parameters. As a result, pixels having similar intensity information but located in different regions can be differentiated. Experimental results shows that, by using DACF, the mass segmentation results in digitized mammograms are improved with optimal mass boundaries, less number of noisy patches, and computational efficiency. An average probability of segmentation error of 7.18% for well-defined masses (or 8.06% for ill-defined masses) was obtained by using DACF on MiniMIAS database, with 5.86% (or 5.55%) and 6.14% (or 5.27%) improvements as compared to the standard DA and fuzzy *c*-means methods.

## 1. Introduction

Image segmentation is a process which divides an image into several meaningful areas such that the segmented image can be further analyzed and interpreted. A segmentation algorithm, in a mammographic context, is an algorithm used to detect something, usually the whole breast or a specific kind of abnormalities like microcalcifications or masses. In the digitized mammograms with low contrast, masses are embedded in various breast tissues with fuzzy margins. This variability introduces a challenge for breast mass segmentation and causes the false positive detection rate to increase as well as decreasing the sensitivity.

In the past decades, a number of image processing techniques have been developed to segment masses from their surrounding breast tissues in digitized mammograms, as reviewed in [1–4]. Among them, clustering methods are one of the most commonly used techniques for image segmentation [5] as well as for mass detection and/or segmentation [4]. Partitioning clustering and hierarchical clustering are two main approaches to clustering. *K*-means [6] and fuzzy *c*-means (FCM) [7] algorithms are widely used partitioning techniques by the researchers in many real world applications. For mass segmentation, *K*-means has been used in [8, 9] to generate initial segmentation results and in [10, 11] to refine an initial detection from adaptive thresholding. FCM was also used for mass segmentation with different objectives: while [12] used it to group pixels with similar grey-level values in the original images, [13] used it over the set of local features extracted from application of a multiresolution wavelet transform and Gaussian Markov random fields analysis. In contrast to *K*-means and FCM, which are sensitive to data initialization and converge to local optimal solutions, deterministic annealing (DA) clustering [14] is a global minimisation algorithm by incorporating randomness into the energy function to be minimized, such that it is independent of the choice of the initial data configuration and has the ability to avoid poor local optima. The DA approach has also been used for mass segmentation in [15, 16].

Most clustering algorithms (including *K*-means, FCM, and DA) perform image segmentation directly from the intensity (or color) space with an intensity filter to enlarge the difference between normal and abnormal breast tissue. The processing time is a prominent advantage of these algorithms. However, the intensity-based methods cannot satisfactorily outline the boundary of the mass region when the image contrast and signal noise ratio are low and therefore lead to poor segmentation results. Markov random field technique was used in mass segmentation [17] to exploit the spatial continuity in order to improve the performance of segmentation algorithm. It has the ability to reduce segmentation error caused by intensity noise; however, the computational cost is high. Reference [18] proposed a fuzzy clustering algorithm incorporating an elliptic shape function for lip image segmentation. The pitfall is that the convergence time increases as the weighting parameter that controls the spatial shape information increases.

In this paper, we propose a novel clustering algorithm based on DA approach to overcome the problems of most existing clustering techniques. In the standard DA clustering [14, 19] for image segmentation, the dissimilarity measure in the objective function is defined merely based on Euclidean distances between the image intensity and the intensity centroids without knowledge of the spatial shape information. Solely using the intensity or intensity related information is hard to differentiate pixels with the same intensity information but located in unconnected regions. As a result, large number of subregions in the same cluster that contains a mass may lead to heavy computational load. Additionally, it is hard to find the fuzzy boundary when the image contrast is low. To handle these challenges, a new dissimilarity measure for DA clustering incorporating a circular shape function (DACF) is proposed. Since both intensity and spatial information are used in the optimization process, the DACF algorithm offers two advantages. First, it is robust against noise and cluster number; that is, pixels having similar intensity information but located in different regions can be differentiated, with just two clusters for the entire images. Second, it is computationally efficient. The convergence time decreases as the difference between the two weighting parameters increases. Experimental results have demonstrated the advantages of the DACF algorithm.

The main contribution of our current work includes the following: (1) the geometry shape is integrated into the intensity feature space for mass segmentation in terms of dynamically fitted circular shape function; (2) the proposed method can differentiate the pixels with the same intensity values but located in different (mass and nonmass) regions, which cannot be achieved by standard clustering methods like FCM and DA; (3) the proposed method achieves better segmentation performance than FCM and DA in terms of segmentation accuracy and computational time; (4) the proposed method is general, which can be integrated into other segmentation algorithms and applicable for other biomedical applications.

The rest of this paper is organized as follows.  Section 2 briefly reviews the standard DA clustering approach and derives the formulation and implementation of the proposed DACF algorithm. The experimental results and related discussions on real mass images are given qualitatively and quantitatively in Section 3. The conclusion is given in Section 4.

## 2. The Proposed Method

### 2.1. A Brief Review of Standard Deterministic Annealing Approach

Suppose there are *l* input vectors **x**_*i*_ ∈ *R* ^*N*^, *i* = 1,…, *l*, which are partitioned into *K* clusters with mass center at {**v**_1_, **v**_2_,…, **v**_*K*_} ⊂ *R* ^*N*^. The DA clustering algorithm [14, 19] aims to minimize the following Lagrangian formulation:

(1) F=D−THs=∑j=1l ∑k=1Kp(xj)p(vk ∣ xj)d(xj,vk)hhhhhhhl−T−∑j=1l ∑k=1Kpxjp(vk ∣ xj)log⁡p(vk ∣ xj),

where *T* is the Lagrange multiplier, which is analogous to the temperature in statistical mechanics, *D* is the cost function, *H*_*s*_ is the Shannon entropy, *p*(**x**_*j*_) is the source distribution (equal to 1/*l* in [19]), *p*(**v**_*k*_∣**x**_*j*_) is the association probability (distribution) relating input point **x**_*j*_ with cluster center **v**_*k*_, and *d*(**x**_*j*_, **v**_*k*_) is the squared Euclidian distance between **x**_*j*_ and **v**_*k*_ defined by

(2) $$\begin{array}{c} d\left(x_{j},v_{k}\right)={\left\|x_{j}-v_{k}\right\|}^{2}. \end{array}$$

It turns out [19] that the resultant distribution is the titled distribution given by

(3) $$\begin{array}{c} p\left(v_{k}\;|\;x_{j}\right)=\frac{p\left(v_{k}\right)e^{-d\left(x_{j},v_{k}\right)/T}}{Z_{x_{j}}}, \end{array}$$

where *Z*_**x**_*j*__ = ∑_*k*=1_^*K*^ *p*(**v**_*k*_)*e* ^−*d*(**x**_*j*_, **v**_*k*_)/*T*^ is the partition function and *p*(*v*_*k*_) = ∑_*j*=1_^*l*^ *p*(**x**_*j*_)*p*(**v**_*k*_∣**x**_*j*_) is the mass probability of *k*(th) cluster. Plugging (3) back into (1), the effective cost to be minimized becomes the free energy (a well-known concept in statistical mechanics [14]) as follows:

(4) $$\begin{array}{c} F^{*}=-T\overset{l}{\underset{j=1}{\sum }}p\left(x_{j}\right)\log\overset{K}{\underset{k=1}{\sum }}p\left(v_{k}\right)e^{-d\left(x_{j},v_{k}\right)/T}. \end{array}$$

The expression of cluster center is then derived by minimizing (4) with respect to *v*_*k*_; that is

(5) $$\begin{array}{c} v_{k}=\frac{\sum_{j=1}^{l}p\left(x_{j}\right)p\left(v_{k}\;|\;x_{j}\right)x_{j}}{\sum_{j=1}^{l}p\left(x_{j}\right)p\left(v_{k}\;|\;x_{j}\right)}. \end{array}$$

Alternatively updating (3) and (5) with phase transition gives the DA algorithm. The DA approach to clustering has demonstrated to be independent of the data initialization and has ability to avoid poor local optima, as discussed in [14, 19].

### 2.2. Deterministic Annealing Clustering Incorporating Circular Function (DACF)

Consider an image with *M* × *N* pixels, whose locations are denoted by (*i*, *j*), where *i* ∈ [1, *M*] and *j* ∈ [1, *N*]. Let us define the new dissimilarity measure ${\hat{d}}_{k,i,j}$ of the proposed DACF by

(6) $$\begin{array}{c} {\hat{d}}_{k,i,j}=d_{k,i,j}+\beta_{k}f\left(k,i,j,s\right), \end{array}$$

where *β*_*k*_ are predefined regulated parameter, *d*_*k*,*i*,*j*_ stands for the Euclidean distance between the (*i*, *j*)th pixel **x**_*i*,*j*_ and the centroid **v**_*k*_ of the *k*th cluster, as

(7) $$\begin{array}{c} d_{k,i,j}={\left\|x_{i,j}-v_{k}\right\|}^{2} \end{array}$$

and *f*(*k*, *i*, *j*, **s**) represents the shape information, given by circular function as

(8) $$\begin{array}{c} f\left(k,i,j,s\right)={\left(i-x_{c}\right)}^{2}+{\left(j-y_{c}\right)}^{2}, \end{array}$$

where **s** = {*x*_*c*_, *y*_*c*_} is a unique clique, and *x*_*c*_, *y*_*c*_ are the physical *X*-*Y* coordinate of the center of a mass region. The dissimilarity measure ${\hat{d}}_{k,i,j}$ consists of a measure of the intensity dissimilarity between the (*i*, *j*)th pixel and the centroid **v**_*k*_ in the intensity feature space, and the spatial distance between the pixel (located at (*i*, *j*)) and the center (denoted by (*x*_*c*_, *y*_*c*_)) of the targeted mass region. With the inclusion of circular shape information, the pixels with similar intensity but located in disjointed region will be differentiated. The purpose of the inclusion of the shape function is to obtain a large membership for the cluster associated with mass region. In order to achieve it, the weighting parameter *β*_*k*_ is defined as the weight of the spatial distance against the intensity feature. According to the dissimilarity definition of the Euclidean distance, the closer a pixel belongs to a cluster, the smaller the distance is. Therefore, the shape distance between the location of a pixel and a specific cluster center is small if the pixel belongs to the cluster; otherwise the distance is larger if it belongs to other clusters.

The expected distortion or objective function of the DACF incorporating spatial information is then defined as

(9) D^=∑i=1M ∑j=1N ∑k=1Kp(xi,j,vk)d^k,i,j=∑i=1M ∑j=1Np(xi,j)∑k=1Kp(vk ∣ xi,j)d^k,i,j=∑i=1M ∑j=1Np(xi,j)∑k=1Kp(vk ∣ xi,j)(dk,i,j+βkf(k,i,j,s))=D+D′,

where *D* is the distortion measure as in the original DA method defined by

(10) $$\begin{array}{c} D=\overset{M}{\underset{i=1}{\sum }}\;\overset{N}{\underset{j=1}{\sum }}p\left(x_{i,j}\right)\overset{K}{\underset{k=1}{\sum }}p\left(v_{k}\;|\;x_{i,j}\right)\left(d_{k,i,j}\right) \end{array}$$

and *D*′ is the distortion measure of spatial information, as

(11) $$\begin{array}{c} D^{\prime }=\overset{M}{\underset{i=1}{\sum }}\;\overset{N}{\underset{j=1}{\sum }}p\left(x_{i,j}\right)\overset{K}{\underset{k=1}{\sum }}p\left(v_{k}\;|\;x_{i,j}\right)\left(\beta_{k}f\left(k,i,j,s\right)\right). \end{array}$$

We recast the optimization problem as seeking the distribution which minimizes $\hat{D}$ subject to a specified level of randomness that is measured by Shannon entropy

(12) $$\begin{array}{c} H_{s}=-\overset{M}{\underset{i=1}{\sum }}\;‍\overset{N}{\underset{j=1}{\sum }}‍\;\overset{K}{\underset{k=1}{\sum }}p\left(x_{i,j},v_{k}\right)\log p\left(x_{i,j},v_{k}\right). \end{array}$$

The optimization is reformulated as minimization of the Lagrangian

(13) F^=D^−THs=D−THs+D′=F+D′.

Minimizing $\hat{F}$ with respect to the probability of *p*(**v**_*k*_∣**x**_*i*,*j*_) leads to the titled distribution [14, 19]

(14) $$\begin{array}{c} p\left(v_{k}\;|\;x_{i,j}\right)=\frac{p\left(v_{k}\right)\exp\left(-{\hat{d}}_{k,i,j}/T\right)}{Z_{x_{i,j}}}, \end{array}$$

where the normalized factor is given by

(15) $$\begin{array}{c} Z_{x_{i,j}}=\overset{K}{\underset{k=1}{\sum }}p\left(v_{k}\right)\exp\left(-\frac{{\hat{d}}_{k,i,j}}{T}\right). \end{array}$$

Taking the partial derivative on $\hat{F}$ with respect to cluster center, we have

(16) $$\begin{array}{c} \frac{\partial \hat{F}}{\partial v_{k}}=\frac{\partial F}{\partial v_{k}}+\frac{\partial D^{\prime }}{\partial v_{k}}=\frac{\partial F}{\partial v_{k}}. \end{array}$$

It can be seen that the partial derivative of the objective function with the new dissimilarity measure with respect to *v*_*k*_ is identical to that of DA. Hence, the formula for computing centroids of DACF in the intensity feature space is the same as in DA; that is,

(17) $$\begin{array}{c} v_{k}=\frac{\sum_{i=1}^{M}\sum_{j=1}^{N}p\left(x_{i,j}\right)p\left(v_{k}\;|\;x_{i,j}\right)x_{i,j}}{\sum_{i=1}^{M}\sum_{j=1}^{N}p\left(x_{i,j}\right)p\left(v_{k}\;|\;x_{i,j}\right)}. \end{array}$$

The partial derivative of $\hat{F}$ with respect to **s** is given by

(18) ∂F^∂s=∂F∂s+∂D′∂s=∂D′∂s=∑i=1M ∑j=1Npxi,j∑k=1Kpvkxi,jβk∂fk,i,j,s∂s;

that is,

(19) $$\begin{array}{c} \frac{\partial \hat{F}}{\partial x_{c}}=\overset{M}{\underset{i=1}{\sum }}\;\overset{N}{\underset{j=1}{\sum }}p\left(x_{i,j}\right)\overset{K}{\underset{k=1}{\sum }}p\left(v_{k}\;|\;x_{i,j}\right)\beta_{k}\frac{\partial f\left(k,i,j,s\right)}{\partial x_{c}}=0,\\ \frac{\partial \hat{F}}{\partial y_{c}}=\overset{M}{\underset{i=1}{\sum }}\;\overset{N}{\underset{j=1}{\sum }}p\left(x_{i,j}\right)\overset{K}{\underset{k=1}{\sum }}p\left(v_{k}\;|\;x_{i,j}\right)\beta_{k}\frac{\partial f\left(k,i,j,s\right)}{\partial y_{c}}=0. \end{array}$$

Substituting (8) into (19), the spatial parameters can be obtained as

(20) $$\begin{array}{c} x_{c}=\frac{\sum_{i=1}^{M}\sum_{j=1}^{N}ip\left(x_{i,j}\right)\sum_{k=1}^{K}\beta_{k}p\left(v_{k}\;|\;x_{i,j}\right)}{\sum_{i=1}^{M}\sum_{j=1}^{N}p\left(x_{i,j}\right)\sum_{k=1}^{K}\beta_{k}p\left(v_{k}\;|\;x_{i,j}\right)},\\ y_{c}=\frac{\sum_{i=1}^{M}\sum_{j=1}^{N}jp\left(x_{i,j}\right)\sum_{k=1}^{K}\beta_{k}p\left(v_{k}\;|\;x_{i,j}\right)}{\sum_{i=1}^{M}\sum_{j=1}^{N}p\left(x_{i,j}\right)\sum_{k=1}^{K}\beta_{k}p\left(v_{k}\;|\;x_{i,j}\right)}. \end{array}$$

Alternatively updating *p*(**v**_*k*_∣**x**_*i*,*j*_) and **v**_*k*_ according to (14) and (17) as well as *x*_*c*_ and *y*_*c*_ according to (20) gives the proposed DACF algorithm.

The titled distribution (14) is the membership of each pixel belonging to different clusters. Generally, the intensities of the center part of a mass region are higher than those locating outside of mass region. For pixels inside a mass region, the intensity dissimilarity is in dominant position, while spatial information plays a major role in dissimilarity measure for pixels outside the mass region. Therefore, the pixels with the same intensity values but locate in different positions in an image will be differentiated, which makes DACF yield better performance for both mass and nonmass related regions.

## 3. Experimental Results

Thirty-six mammograms from MiniMIAS database [20] that contain thirty-nine masses with various backgrounds (fatty, fatty glandular, and dense-glandular breast tissues) were examined. The mammograms mdb005, mdb132, and mdb144 each contain two mass regions. The two masses in mammogram mdb005 were heavily overlapped, so they were processed together as a single one. The two mass regions in mdb132 and mdb144 were processed independently. Therefore, thirty-eight regions of interest (ROIs) were analyzed. Instead of automatic extraction, in this study, the ROIs were taken from the mammographic image based on the information provided by the database. The size of each extracted ROI, as well as the center and radius of each mass are listed in the appendix at the end of this paper.

**Figure 1:**
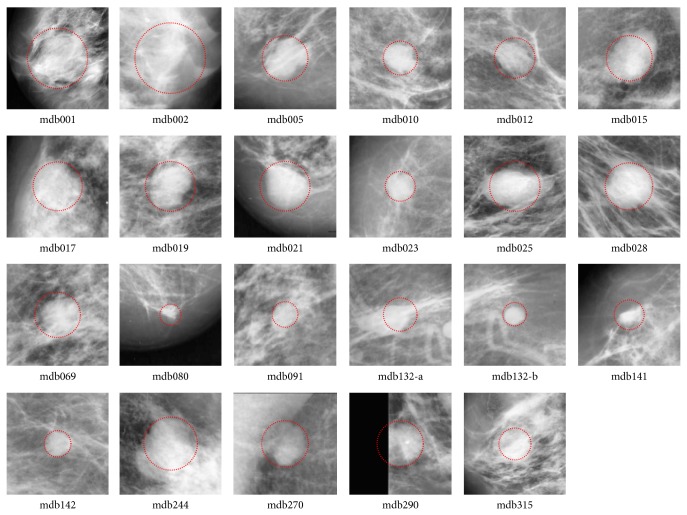
The twenty-three ROIs that contain masses with well-defined shape in MiniMIAS database. The two ROIs in mdb132 are processed independently. Gaussian filter and image equalization are used to phase out noisy points and enhance mass regions.

**Figure 2:**
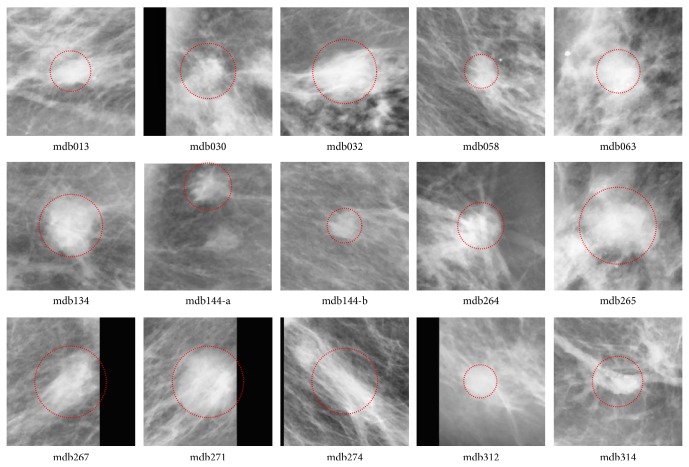
The fifteen ROIs that contain masses with ill-defined shape in MiniMIAS database. The two ROIs in mdb144 are processed independently. Gaussian filter and image equalization are used to phase out noisy points and enhance mass regions.

According to the information of “class of abnormality” provided by the database, the thirty-eight ROIs were classified into two categories: well-defined masses (twenty-three cases) and ill-defined masses (fifteen cases). The ROIs including well-defined masses are illustrated in Figure 1, while the ROIs including ill-defined masses are shown in Figure 2. Gaussian filter (kernel size 3 × 3 and standard deviation 1.0) and image equalization are used to phase out noisy points and enhance mass regions in the image preprocessing step. In all examples, we fix the fuzziness degree *m* = 2 for the FCM algorithm, and the annealing factor alpha = 0.9 for the standard DA and proposed DACF algorithms.

### 3.1. Segmentation Results

Three clustering methods of DACF, DA, and FCM are tested on the twenty-three ROIs with well-defined masses and fifteen ROIs with ill-defined masses to show their performance on mammographic mass segmentation. The decision to choose the cluster that contains mass region is based on the assumption that the suspicious mass area is brighter than its surrounding breast tissues, which is valid for most of the real applications [17]. For the illustration purpose, the clustering results are transformed into binary images, where pixels with gray value 128 belong to the suspicious cluster, and pixels with gray value 0 belong to the nonmass cluster.

**Figure 3:**
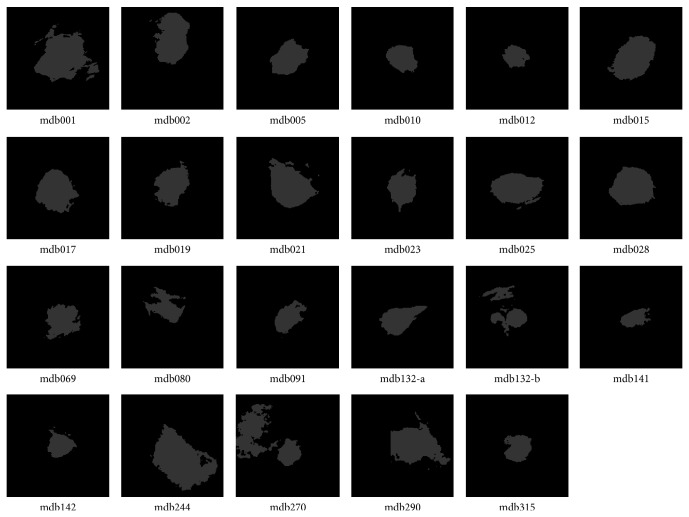
Segmentation results by DACF for the twenty-three ROIs in Figure 1, where the pixels in the white region belong to the mass cluster, and pixels in dark region belong to the nonmass cluster.

**Figure 4:**
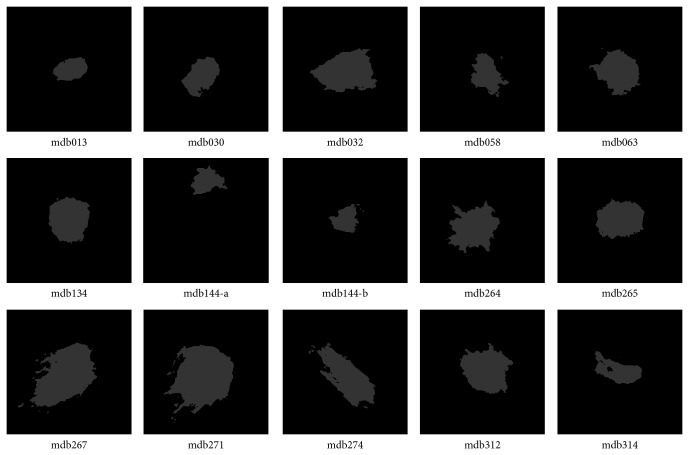
Segmentation results by DACF for the fifteen ROIs in Figure 2, where the pixels in the white region belong to the mass cluster, and pixels in dark region belong to the nonmass cluster.

**Figure 5:**
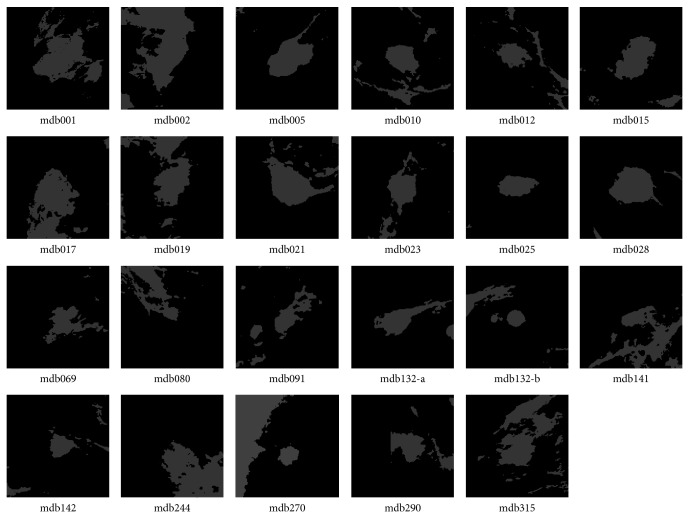
Segmentation results by DA for the twenty-three ROIs in Figure 1, where the pixels in the white region belong to the mass cluster, and pixels in dark region belong to the nonmass cluster.

**Figure 6:**
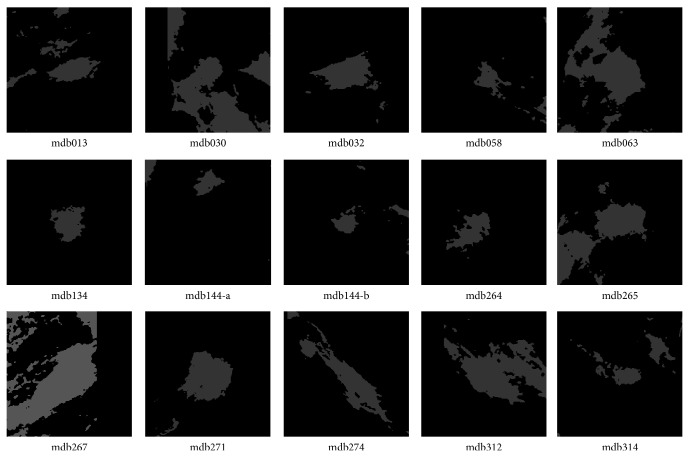
Segmentation results by DA for the fifteen ROIs in Figure 2, where the pixels in the white region belong to the mass cluster, and pixels in dark region belong to the nonmass cluster.

**Figure 7:**
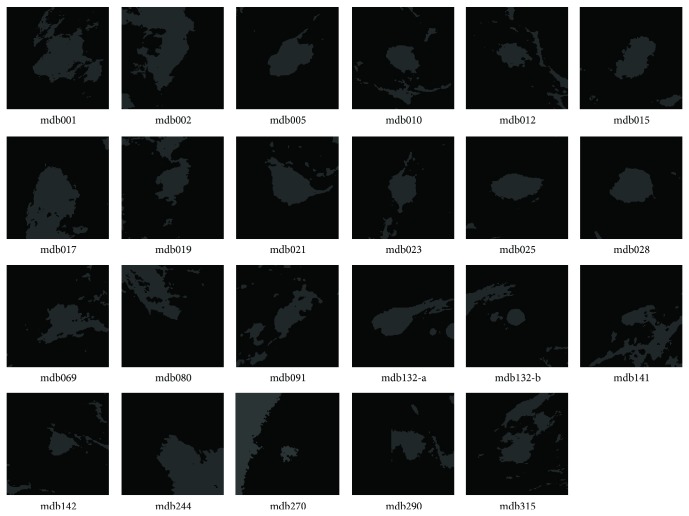
Segmentation results by FCM for the twenty-three ROIs in Figure 1, where the pixels in the white region belong to the mass cluster, and pixels in dark region belong to the nonmass cluster.

**Figure 8:**
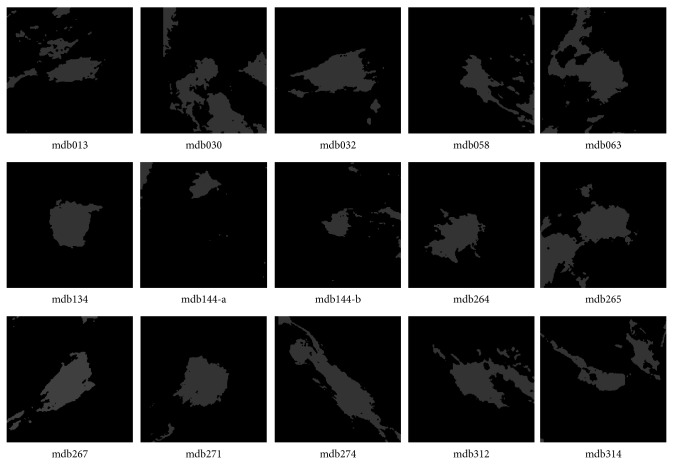
Segmentation results by FCM for the fifteen ROIs in Figure 2, where the pixels in the white region belong to the mass cluster, and pixels in dark region belong to the nonmass cluster.

In the experiment, the cluster number is set as two for DACF and two to six for DA and FCM (in order to get reasonable results). It is one of the advantages of DACF to use the fixed cluster number (two in our experiment). The values of weighting parameter *β*_1_ = 1/4 (for mass region) and *β*_2_ = 10 (for background) were applied to the testing dataset; the details of the value selection can be found in the next subsection. Figures 3 and 4 show the segmentation results by the DACF algorithm for the ROIs in Figures 1 and 2, respectively, where pixels with high intensity (gray value 128) belong to the suspicious cluster. The mass region in each ROI is identified as the one with the maximum number of pixels in the suspicious cluster. Figures 5 and 6 show the segmentation results by DA, and the segmentation results by FCM are illustrated in Figures 7 and 8, respectively. From the figures, it can be seen that due to the incorporation of spherical shape information, pixels belonging to the same intensity feature cluster while locating in different positions can be differentiated to certain degrees by DACF. In contrast, standard DA and FCM failed to differentiate them in most cases. Additionally, less number of patched regions was found in the mass cluster by DACF shown in Figures 3 and 4, as compared to that of the standard DA shown in Figures 5 and 6 and FCM shown in Figures 7 and 8.

In order to evaluate the segmentation performance, a quantitative technique was applied to the three clustering algorithms on mammographic masses. The methods used to evaluate the quality of image segmentation algorithms can be broadly classified into two groups, supervised and unsupervised approaches. Unsupervised evaluation does not depend on a true segmentation [21], while in supervised evaluation, the difference between a reference segmentation and the output of a segmentation algorithm is computed. (Unsupervised evaluation is stand-alone and objective, which does not request any user intervention. But we will use supervised evaluation in our work due to the following: (1) one of the issues we have to consider is that the unsupervised method may not perform well in comparison evaluation produced by different algorithms and in comparing human versus machine segmentations [22]; (2) another consideration is, in the field of biomedical image analysis, it is common to use supervised but not unsupervised method for the evaluation of image segmentation.) This study chose a supervised evaluation method and the mass boundaries were given by the physician. The probability of segmentation error (PSE) is formulated by [23]

(21) $$\begin{array}{c} \text{PSE}=P\left(O\right)P\left(B\;|\;O\right)+P\left(B\right)P\left(O\;|\;B\right), \end{array}$$

where *P*(*O*) and *P*(*B*) are the priori probability of the object (mass region) or background (nonmass region), respectively. *P*(*B*∣*O*) is the probability of classifying objects as background, and *P*(*O*∣*B*) is the probability of classifying background as object. Suppose the pixel number of the mass region in the reference segmentation image is *N*_trueobj_ and the pixel number of the mass region in the segmented image by DACF, DA, or FCM is *N*_calobj_, then the probabilities are defined as

(22) P(B ∣ O)=Ntrueobj−Rtrueobj∩RcalobjNtrueobj,P(O ∣ B)=Ncalobj−Rtrueobj∩RcalobjNcalobj,

where *R*_trueobj_ is the region of mass in reference image, and *R*_calobj_ is the calculated mass region by DACF, DA, and FCM. Therefore, *R*_trueobj_∩*R*_calobj_ represent the number of pixels in the overlapped mass region between the reference image and calculated image. The computed PSE for DACF, DA, and FCM for the ROIs in Figures 1 and 2 are shown in Tables 1 and 2, respectively. We can see that DACF performs better than DA and FCM algorithms for almost all the ROIs, especially for the cases with comparatively low contrast such as mdb091 and mdb141 for well-defined masses and mdb030 and mdb063 for ill-defined masses. Numerically, for the well-defined cases, an average PSE of 7.18% was obtained by using DACF, as compared to 13.04% and 13.32% by using standard DA and FCM methods, respectively; while for the ill-defined cases, DACF achieved an average PSE of 8.06%, with 5.55% and 5.27% improvement when compared with standard DA and FCM methods, respectively.

### 3.2. Discussions

#### 3.2.1. Weighting Parameter Analysis

The weighting parameter *β* in (6) controls the influence of the geometrical distance and the intensity feature in the dissimilarity measure. It is desirable that the membership of a pixel is close to one if it is located near the center of a mass. For a pixel far away from the center of the mass, its membership to the cluster should be close to zero. Suppose that the two clusters in DACF algorithm are cluster 1 and cluster 2, where cluster 1 represents the mass region and cluster 2 represents the nonmass region. For an intensity-based algorithm, like standard DA or FCM, the membership of a pixel in nonmass region to cluster 1 may approximately equal one if it has the same intensity value as the pixels in mass region. In this situation, it is hoped that the spatial information will be dominant in the objective function of DACF; that is, the weighting parameter of cluster 2 should be large enough such that the influence of intensity-based feature is reduced significantly. For the weighting parameter of cluster 1, it is expected that the *β* value should be small enough such that the intensity-based feature is dominant in the objective function.

**Figure 9:**
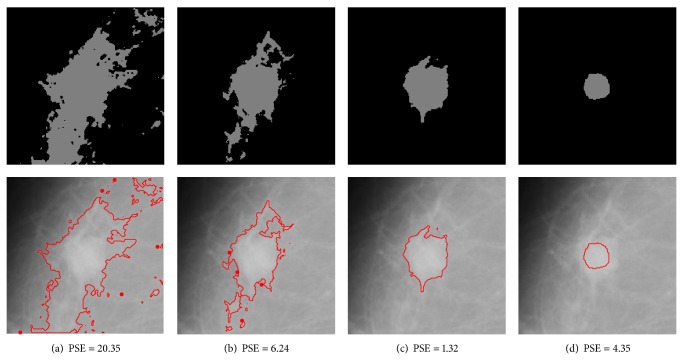
Segmentation results for case mdb023 (with fatty-glandular breast tissues) using DACF with different *β*_1_ and *β*_2_ parameters. The lowest PSE is achieved at *β*_1_ = 1/4, *β*_2_ = 10. Top row: binary images after DACF segmentation. Bottom row: overlay images consisting of the segmented mass boundaries overlayed over the raw input images. (a) *β*_1_ = 1, *β*_2_ = 1. (b) *β*_1_ = 1/2, *β*_2_ = 5. (c) *β*_1_ = 1/4, *β*_2_ = 10. (d) *β*_1_ = 1/8, *β*_2_ = 20.

**Figure 10:**
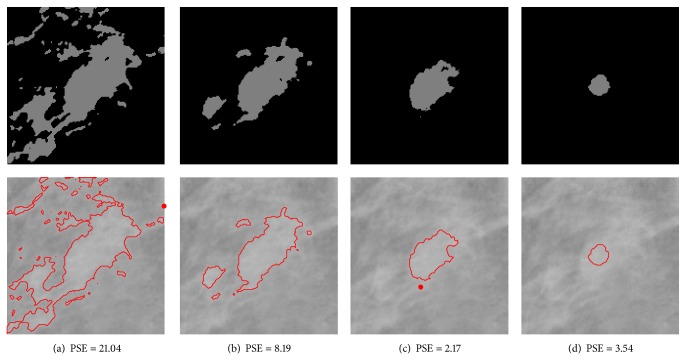
Segmentation results for case mdb091 (with fatty breast tissues) using DACF with different *β*_1_ and *β*_2_ parameters. The lowest PSE is achieved at *β*_1_ = 1/4, *β*_2_ = 10. Top row: binary images after DACF segmentation. Bottom row: overlay images consisting of the segmented mass boundaries overlayed over the raw input images. (a) *β*_1_ = 1, *β*_2_ = 1. (b) *β*_1_ = 1/2, *β*_2_ = 5. (c) *β*_1_ = 1/4, *β*_2_ = 10. (d) *β*_1_ = 1/8, *β*_2_ = 20.

**Figure 11:**
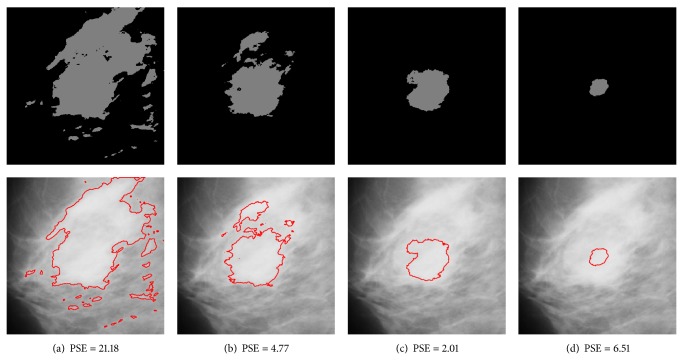
Segmentation results for case mdb315 (with dense-glandular breast tissues) using DACF with different *β*_1_ and *β*_2_ parameters. The lowest PSE is achieved at *β*_1_ = 1/4, *β*_2_ = 10. Top row: binary images after DACF segmentation. Bottom row: overlay images consisting of the segmented mass boundaries overlayed over the raw input images. (a) *β*_1_ = 1, *β*_2_ = 1. (b) *β*_1_ = 1/2, *β*_2_ = 5. (c) *β*_1_ = 1/4, *β*_2_ = 10. (d) *β*_1_ = 1/8, *β*_2_ = 20.

However, it is difficult to analyze the two weighting parameters separately since the incorporation of different values of *β*_1_ and *β*_2_ will lead to different dissimilarity measures. According to the experimental results, we find that the influence of weighting parameter on the segmentation results depends on the image content of each ROI. Experimental results show that better segmentation results are obtained when the value of *β*_1_ is smaller than one, and the value of *β*_2_ is relatively large. In order to illustrate the relationship between the values of the weighting parameters and the segmentation results, experiments are carried out on three ROIs that contain mass with different kinds of backgrounds: mdb023 with fatty-glandular breast tissues, mdb091 with fatty breast tissues, and mdb315 with dense-glandular breast tissues. The segmentation results are shown in Figures 9, 10, and 11 for each ROI. The PSE is also provided to evaluate the segmentation results under different values of weighting parameters. It can be seen from the figures that the values of *β*_1_ = 1/4 and *β*_2_ = 10 are optimal values for these three ROIs.

#### 3.2.2. Convergence Time Analysis

The main parameter that affects the convergence time in DACF is the weighting parameter since the number of clusters was fixed (two clusters were used). The convergence time here refers to the total CPU time for clustering. A Duo Core 1.59 GHz laptop with MATLAB 8 was used to run the three clustering algorithms. For DA and FCM, the relative segmentation results were obtained with a wide range of number of clusters from two to six, in order to get reasonable results.  Table 3 shows the convergence time of DACF, DA, and FCM for the segmentation results as shown in Figures 3, 5, and 7, respectively while Table 4 shows the convergence time of DACF, DA, and FCM for the segmentation results as shown in Figures 4, 6, and 8, respectively. It can be seen that DACF has less convergence time than DA and FCM, while FCM runs faster as compared to DA.

The convergence speed of DACF is not affected significantly by changing the values of weighting parameters. Basically, the convergence time decreases or keeps approximately constant as the difference between the two weighting parameters increases. Through experiments, it can be seen that the relatively higher difference between the two weighting parameters makes the DACF capable of handling images with more complicated content. In contrast, to handle this situation, larger number of clusters has to be used for both DA and FCM to obtain reasonable segmentation results.

## 4. Conclusion

The proposed DACF algorithm offers two advantages for mammographic mass segmentation on extracted ROIs. First, the segmentation ability is improved. The average PSE by DACF is much smaller than those by standard DA and FCM. Additionally, less number of patched regions was found in mass cluster by using DACF. Second, the convergence time is reduced. In DACF, the number of clusters is two, and the optimal segmentation results were obtained by regulating the weighting parameters, with much less convergence time for all the thirty-eight cases as compared to those by DA and FCM. To summarize, DACF is robust against noisy regions and computationally efficient with a fixed number of clusters. Unlike classical clustering methods for image segmentation, the objective function of DACF contains both intensity-based information and geometry-based circular shape function as a means to improve the image data partitions. Experimental results show that the proposed DACF improved the segmentation performance for mammographic images.

It is noted that one of the major limitations of the proposed method is that the current formulation can only deal with two clusters. We will investigate the possibility to incorporate multicircular shape to handle more general cases in the near future. It is also noted that the current study determines the weighting parameter *β* through experiments; though it works for all the tested cases in this paper, a numerical solution is desirable to improve the intelligence of the proposed method. There is a need to verify the efficiency of the proposed method by performing evaluations on other mammographic image sets, such as the DDSM database [24]. These will be the subjects of our future research on mammographic mass segmentation. What is more, it is worthy to mention that the proposed approach is general, which may be applicable for other biomedical applications like left ventricle segmentation from cardiac magnetic resonance images. We will also investigate this topic in our future research work.

## Figures and Tables

**Figure 12:**
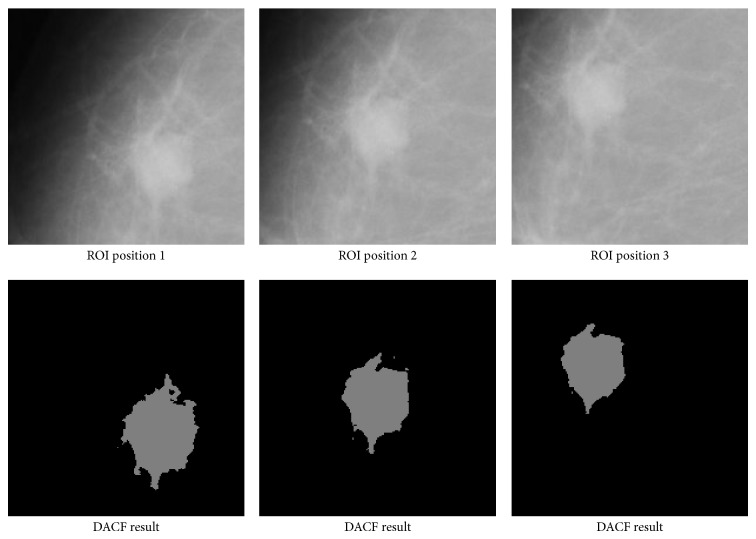
The proposed DACF successfully segments the mass with different ROI shifting, though the final results may be a bit different.

**Figure 13:**
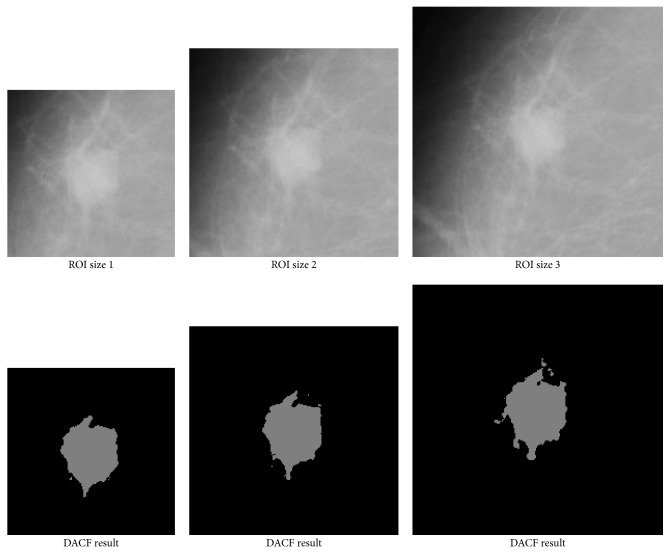
The proposed DACF successfully segments the mass with different ROI size, though the final results may be a bit different.

**Table 1:** The probability of segmentation error (PSE) by DACF, DA, and FCM on twenty-three ROIs with well-defined masses in Figure 1.

Image	PSE of DACF	PSE of DA	PSE of FCM
	(%)	(%)	(%)
mdb001	4.87	11.70	12.12
mdb002	28.88	32.58	32.15
mdb005	3.40	6.94	6.27
mdb010	2.61	9.33	8.2
mdb012	8.43	12.97	12.49
mdb015	5.18	7.30	6.83
mdb017	6.38	16.00	17.03
mdb019	9.48	19.17	17.50
mdb021	6.71	11.21	10.46
mdb023	1.31	4.58	3.33
mdb025	4.65	3.65	4.79
mdb028	4.47	6.42	6.20
mdb069	7.42	11.12	12.34
mdb080	2.57	12.32	15.61
mdb091	2.17	7.69	10.51
mdb132-a	4.25	7.75	10.12
mdb132-b	5.16	7.92	8.67
mdb141	3.67	18.55	17.93
mdb142	1.68	4.81	5.79
mdb244	17.25	29.69	30.36
mdb270	21.45	29.67	32.87
mdb290	10.99	12.20	12.19
mdb315	2.24	16.25	12.62

Average of PSE	7.18	13.04	13.32

**Table 2:** The probability of segmentation error (PSE) by DACF, DA, and FCM on fifteen ROIs with ill-defined masses in Figure 2.

Image	PSE of DACF	PSE of DA	PSE of FCM
	(%)	(%)	(%)
mdb013	3.67	7.16	9.03
mdb030	9.05	27.63	25.84
mdb032	7.74	10.70	10.64
mdb058	3.15	5.43	7.76
mdb063	2.23	15.90	12.48
mdb134	9.34	8.94	8.11
mdb144-a	14.08	14.10	14.51
mdb144-b	2.47	4.42	4.54
mdb264	6.78	8.55	9.53
mdb265	18.83	26.57	26.58
mdb267	10.51	18.32	15.57
mdb271	8.15	12.70	12.49
mdb274	11.76	19.87	20.15
mdb312	5.93	16.33	9.12
mdb314	7.23	11.77	13.56

Average of PSE	8.06	13.61	13.33

**Table 3:** The convergence time of DACF, DA, and FCM for the segmentation results in Figures 3, 5, and 7, respectively.

Image	DACF (sec)	DA (sec)	FCM (sec)
mdb001	2.78	14.77	9.62
mdb002	0.72	3.27	2.74
mdb005	0.62	2.35	2.19
mdb010	0.58	1.90	1.52
mdb012	0.47	2.37	1.98
mdb015	0.48	2.51	1.84
mdb017	0.48	3.05	2.27
mdb019	0.41	2.10	1.71
mdb021	0.41	5.55	1.07
mdb023	0.67	1.85	1.66
mdb025	0.84	3.35	2.05
mdb028	0.72	3.29	2.63
mdb069	0.58	2.01	1.57
mdb080	0.53	3.90	1.49
mdb091	0.33	1.76	0.53
mdb132-a	0.25	0.82	0.60
mdb132-b	0.31	0.68	0.49
mdb141	0.72	2.80	2.83
mdb142	0.50	2.30	1.26
mdb244	0.39	3.96	1.23
mdb270	0.31	1.43	0.95
mdb290	0.58	3.88	2.43
mdb315	2.06	11.85	7.98

**Table 4:** The convergence time of DACF, DA, and FCM for the segmentation results in Figures 4, 6, and 8, respectively.

Image	DACF (sec)	DA (sec)	FCM (sec)
mdb013	0.42	3.79	1.11
mdb030	1.33	3.43	1.42
mdb032	0.39	4.21	1.85
mdb058	0.25	1.93	1.19
mdb063	0.41	2.23	1.22
mdb134	0.42	2.51	1.87
mdb144-a	0.37	1.48	0.94
mdb144-b	0.33	1.15	1.69
mdb264	0.50	1.45	1.07
mdb265	0.41	1.71	1.78
mdb267	0.59	0.79	1.09
mdb271	0.55	1.49	1.86
mdb274	0.66	1.71	2.91
mdb312	0.50	1.24	0.97
mdb314	0.31	2.09	1.40

**Table 5:** The related information for each extracted ROI.

Image	ROI size	ROI/mass center	Mass radius	Image	ROI size	ROI/mass center	Mass radius
(well-defined)	(pixel)	(*x*, *y*)	(pixel)	(ill-defined)	(pixel)	(*x*, *y*)	(pixel)
mdb001	200	(535, 599)	131	mdb013	100	(667, 659)	31
mdb002	100	(522, 744)	69	mdb030	100	(322, 348)	43
mdb005	100	(488, 873)	45	mdb032	100	(388, 282)	49
mdb010	100	(525, 599)	33	mdb058	100	(318, 665)	27
mdb012	100	(471, 566)	40	mdb063	100	(546, 561)	33
mdb015	100	(595, 160)	45	mdb134	100	(469, 296)	49
mdb017	100	(547, 451)	48	mdb144-a^*^	80	(233, 30)	29
mdb019	100	(653, 547)	49	mdb144-b	100	(313, 484)	27
mdb021	100	(493, 899)	49	mdb264	100	(596, 593)	36
mdb023	100	(538, 343)	29	mdb265	100	(593, 526)	60
mdb025	120	(674, 581)	52	mdb267	100	(793, 543)	56
mdb028	120	(338, 710)	56	mdb271	120	(784, 754)	68
mdb069	100	(462, 618)	44	mdb274	120	(127, 519)	61
mdb080	100	(432, 875)	20	mdb312	80	(240, 761)	20
mdb091	80	(680, 530)	20	mdb314	100	(518, 833)	39
mdb132-a	80	(252, 236)	26				
mdb132-b	80	(335, 258)	18				
mdb141	100	(470, 265)	29				
mdb142	100	(347, 388)	26				
mdb244	100	(466, 457)	52				
mdb270	80	(356, 79)	36				
mdb290	100	(337, 671)	45				
mdb315	200	(516, 577)	62				

^*^In the case of mdb144-a, ROI center is not identical to mass center since the latter is too close to image border.

## Appendix

Table 5 lists the related information for each extracted ROIs, that is, the size of ROI, the center of the ROI/mass, and the radius of the mass. (In our implementation, we extract the ROI in terms of a square, with the center identical to the center of mass and the width determined by ROI size. In the case of mdb144-a, ROI center is not identical to mass center since the latter is too close to image border. As demonstrated in Figures 12 and 13, DACF segmentation results are only slightly affected by the shifting and size of the extracted ROI.) The latter two are used for the reference segmentation.
